# Assessment of *TCF7L2* expression after bariatric surgery

**DOI:** 10.1371/journal.pone.0216627

**Published:** 2019-05-13

**Authors:** Carlos Eduardo S. Macedo, Guilherme da Conti, Andriu S. Catena, Danyelly Bruneska, Malu Rosa, Clarissa G. Noronha, Fernando Santa Cruz, Álvaro A. B. Ferraz

**Affiliations:** 1 General Surgery Unit, Hospital das Clínicas, Federal University of Pernambuco, Recife, PE, Brazil; 2 Laboratory of Immunopathology Keizo Asami, Federal University of Pernambuco, Recife, PE, Brazil; 3 Department of Biochemistry, Federal University of Pernambuco, Recife, PE, Brazil; 4 Federal University of Pernambuco School of Medicine, Recife, PE, Brazil; 5 Department of Surgery, Federal University of Pernambuco, Recife, PE, Brazil; Universitatea de Medicina si Farmacie Carol Davila Biblioteca, ROMANIA

## Abstract

**Objective:**

To assess the influence of bariatric surgery on transcription factor 7-like 2 (*TCF7L2*) expression and its association with body mass index (BMI) and Type 2 diabetes mellitus (T2DM).

**Methods:**

Prospective study performed between 2016 and 2018, where 26 obese patients undergoing bariatric surgery were divided into two subgroups: diabetics and non-diabetics. The RNAs were extracted from peripheral blood samples that were obtained from each patient in two different moments: before surgery and after 12 months of follow-up. The relative expression of *TCF7L2* was determined according to the delta-Ct method.

**Results:**

The linear regression model of BMI x delta-Ct showed a positive correlation (p = 0.037). In the subgroups, an inversely proportional relationship was found between delta-Ct and BMI in the diabetic group and a directly proportional relationship in the non-diabetic group (p>0.05 in both). In the postoperative period, the regression model was similar to the preoperative, except when analyzing the subgroups, where diabetic patients showed a directly proportional relationship (p>0.05). The relative expression of *TCF7L2* showed an average of 1.16 ± 0.91, CI-95% 0.79–1.53. There was an increase in relative expression of 48% in the non-diabetic group (p = 0.021), and a decrease of 27% in the T2DM group (p>0.05) in the postoperative. There was a positive correlation between a greater decrease in BMI and increased relative expression (p = 0.027).

**Conclusion:**

Our results showed that generally, the *TCF7L2* expression increase with a decrease in BMI, however, for patients with T2DM, it exhibits an inverse pattern, which is normalized one year after bariatric surgery.

## Introduction

The transcription factor 7-like 2 (*TCF7L2*) belongs to a family of transcription factors that regulates several embryonic development processes and also affects several cell lines and organs in adult individuals [1]. Its product is a high-mobility group (HMG) that includes the transcription factors involved in homeostasis of blood glucose through the Wnt signaling pathway [2]. A strong association was reported between the polymorphisms of *TCF7L2* gene and risk for T2DM, with a population risk of 21% associated to *TCF7L2* [3–6].

In a pioneering study, Hindle et al. investigated *TCF7L2* expression in obese patients who underwent bariatric surgery, analyzing its relationship with the BMI of the study population and later in subgroups of patients according to the presence or absence of T2DM. The linear regression model showed a negative correlation between BMI and normalized genetic expression of *TCF7L2* in diabetic individuals, while the opposite was found for non-diabetic individuals: *TCF7L2* expression increased with increased BMI [7].

The referred study and other subsequent studies have aroused questioning on the role of the change in *TCF7L2* expression in solving resolution of obesity and diabetes observed after bariatric surgery, as well as on the possible relationship between gene expression and BMI and on whether such expression can be affected by the presence or absence of T2DM [8–11].

The present study aimed to prospectively evaluate the behavior of *TCF7L2* gene expression before and after bariatric surgery, to check the correlation between weight loss and *TCF7L2* expression one year after bariatric surgery, and the correlation between BMI and gene expression between diabetic and non-diabetic patients before and after surgery.

## Materials and methods

### Subjects

This was a prospective cohort study performed during the period between 2016–2018, wherein 26 obese patients submitted to bariatric surgery at the *Hospital das Clínicas*, Federal University of Pernambuco (UFPE), were examined. The participants were patients with indication for bariatric surgery (gastric bypass surgery or sleeve gastrectomy) according to the standard NIH criteria, aged between 18 and 65 years of both genders. Exclusion criteria were pregnancy, recent surgery (in last three months), and concomitant inflammatory and/or infectious process.

The considered diagnostic criteria for T2DM were those of the WHO and ADA [12]. The surgical techniques used were laparoscopic Roux-en-Y Gastric Bypass and laparoscopic sleeve gastrectomy.

All procedures performed in this study, which involves human participants, were in accordance to the ethical standards of the institutional research committee and with the 1964 Helsinki declaration and its later amendments or comparable ethical standards. This research project was approved by the Ethics Committee for Research involving human beings of the Federal University of Pernambuco’s Center of Health Sciences under the registration number 431.576. The volunteers signed the free informed consent form prior to their inclusion in the study.

### Collection of peripheral blood

The collection was performed in two stages: before surgery and after 12 months of follow-up. The samples were collected in PAXgene Blood RNA tubes (Qiagen, Germantown, MD) which allow stabilization and purification of the genetic material, contributing to obtain the desirable quality and quantity of intracellular ribonucleic acid (RNA). The samples were immediately packed and sent to the laboratory where they were duly stored at -80°C.

### RNA extraction

Sample extraction was performed in two stages: first, before bariatric surgery; and second, 12 months after surgical intervention. The samples were extracted in an automated manner for RNA purification and isolation using the QIAsymphony (Qiagen, Germantown, MD) system, from blood samples collected in PAXgene (Qiagen) tubes. The samples were processed using QIAsymphony PAXgene Blood RNA Kit (Qiagen), according to the manufacturer’s recommendations.

### RNA quantification

The samples were quantified using a NanoDrop 2,000 spectrophotometer (Thermo Scientific). Quantification was essential to measure the concentration and purity of RNA extracted by the QIAsymphony automated system (Qiagen), using a ratio of ~2.0 as reference value to assess the extraction quality.

### RT-PCR

RT-PCR (reverse transcription polymerase chain reaction) was subsequently conducted for synthesizing complementary Deoxyribonucleic acid (cDNA) of the samples, using a commercial QuantiTect Reverse Transcription Kit (Qiagen). The obtained cDNA was stored at -20°C until the moment of quantification, which was also performed in NanoDrop spectrophotometer (Thermo Scientific), with a ratio of ~1.8 for assessment of purity. β-actin was used as housekeeping gene, or an endogenous reference gene to normalize the expression. The primer sequence of *TCF7L2* for qPCR was: sense– 5’-CACAC TTACC AGCCG ACGTA-3’, antisense– 5’-TCCTG TCGTG ATTGG GTACA-3’. For β-actin, the Hs_ACTB_1_SG QuantiTect Primer Assay (NM_001101) (Qiagen) was used.

### Quantitative real-time PCR (qPCR)

qPCR was performed using a Rotor-Gene SYBR Green PCR Kit (Qiagen) through RotorGene Q (Qiagen) to obtain a final volume of 25 μL of the reaction (40 cycles of 95°C for 5 seconds, 60°C for 10 seconds).

The relative expression levels of the genes assessed during real-time PCR were determined according to the Ct comparative method: Ct (a threshold cycle) was obtained for each sample, which is defined as the fractional cycle number at which the fluorescence passes the fixed threshold. Since gene expression is analyzed in relation to internal control (housekeeping gene), delta-Ct, or the difference between Ct means of each sample and the mean of the internal control Ct (beta-actin) were calculated. The relative expression was subsequently calculated. For calculating the relative abundance of transcripts of each gene in relation to the abundance of transcripts in the sample reference, the formula 2-relative expression was applied, as the calculation method, its derivations and assumptions were published in 2001 and have been used in specialized articles ever since [13].

### Statistical analysis

Statistical analysis was performed using the *Statistical Package for the Social Sciences* (SPSS), version 18. In the assessment of demographic and clinical data of the patients, the percentage frequencies were calculated and the respective frequency distributions were constructed. The Chi-square test was used for comparing the obtained percentages. Assessments of delta-Ct normality and relative expression were performed through the Kolmogorov-Smirnov test, and when normality was indicated, the Student’s T-test was applied to one sample to check for changes in the expression of the assessed group. After quantitative assessment of relative expression, the individuals were classified considering an expression lower than 1 as decreased and higher than 1 as increased. The Chi-square test for independence was used to assess the possible effect of T2DM on the increase or decrease of gene expression. When the test assumption was not met, the p-value was calculated through Fisher’s exact test. Furthermore, linear regression was used in assessing the relationship of BMI with normalized expression (delta-Ct) and the adjustment model for BMI x Expression was estimated based on pre and postoperative data. Lastly, linear regression analysis of decreased BMI x relative expression was performed. A maximum 5% probability of rejecting the null hypothesis was considered in all cases.

## Results

Twenty-six obese patients who have undergone RYGB or SG were included in this study. Most patients were female (92.3%), with a mean age of 38.1±10.8, non-diabetic (57.7%) and diagnosed with class III obesity (76.9%). The mean BMI before surgery was 45.19 Kg/m^2^, with a standard deviation (SD) of 6.42. Glycated hemoglobin (HbA1c) mean levels in the diabetic patients were 6.7% with a SD of 0.9 (Table 1).

**Table 1:** Participant characteristics (n = 26, Mean±SD).

**Sex (Male/Female)**	2/24
**Age (years)**	38.1±10.8
**Preoperative BMI (Kg/m**^**2**^**)**	45.19±6.42
**T2DM**	11
**HbA1c (%)**	6.7±0.9
**Fasting Glucose**	109.0±43.6

**Fig 1:**
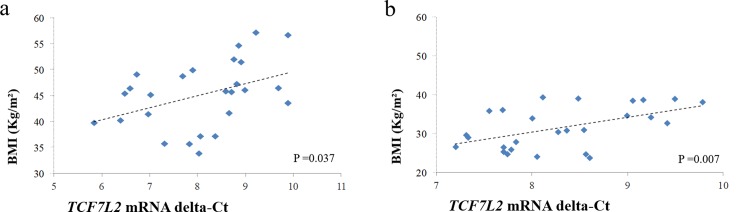
Linear regression analysis: Genetic expression (delta-Ct) *TCF7L2* x BMI (body mass index) in: a) preoperative period and; b) postoperative period (one year after surgery).

Analysis of linear regression models of BMI x delta-Ct showed a positive correlation between BMI and delta-Ct (Fig 1A), in other words it has shown a negative correlation between BMI and genetic expression, and this relationship was statistically significant (p = 0.037).

The same analysis model was used to assess the subgroups of diabetic and non-diabetic patients, where an inverse correlation was found between delta-Ct and BMI in the diabetic group and a positive correlation was found in the non-diabetic group. In other words, it was found a positive correlation between BMI and *TCF7L2* expression among diabetic patients, and an inverse correlation between these same variables in the non-diabetic group. However, this relationship was not significant in both groups (p-value = 0.378 and 0.061, respectively).

The same regression model was used in the postoperative period, and the obtained results were similar to those in the preoperative period: delta-Ct presented a positive correlation with BMI (Fig 1B). However, in the assessment of the subgroups of diabetic and non-diabetic patients in the postoperative period, and unlike the findings for the preoperative period, both groups showed a positive correlation between delta-Ct and BMI, with statistical significance (p = 0.039) only in the non-diabetic group.

The relative expression of *TCF7L2* showed an average of 1.16, standard deviation of 0.91, CI-95% 0.79–1.53. Thus, there was an increase of 16% in the *TCF7L2* gene expression one year after surgery, but this difference was not statistically significant (p = 0.381).

**Fig 2:**
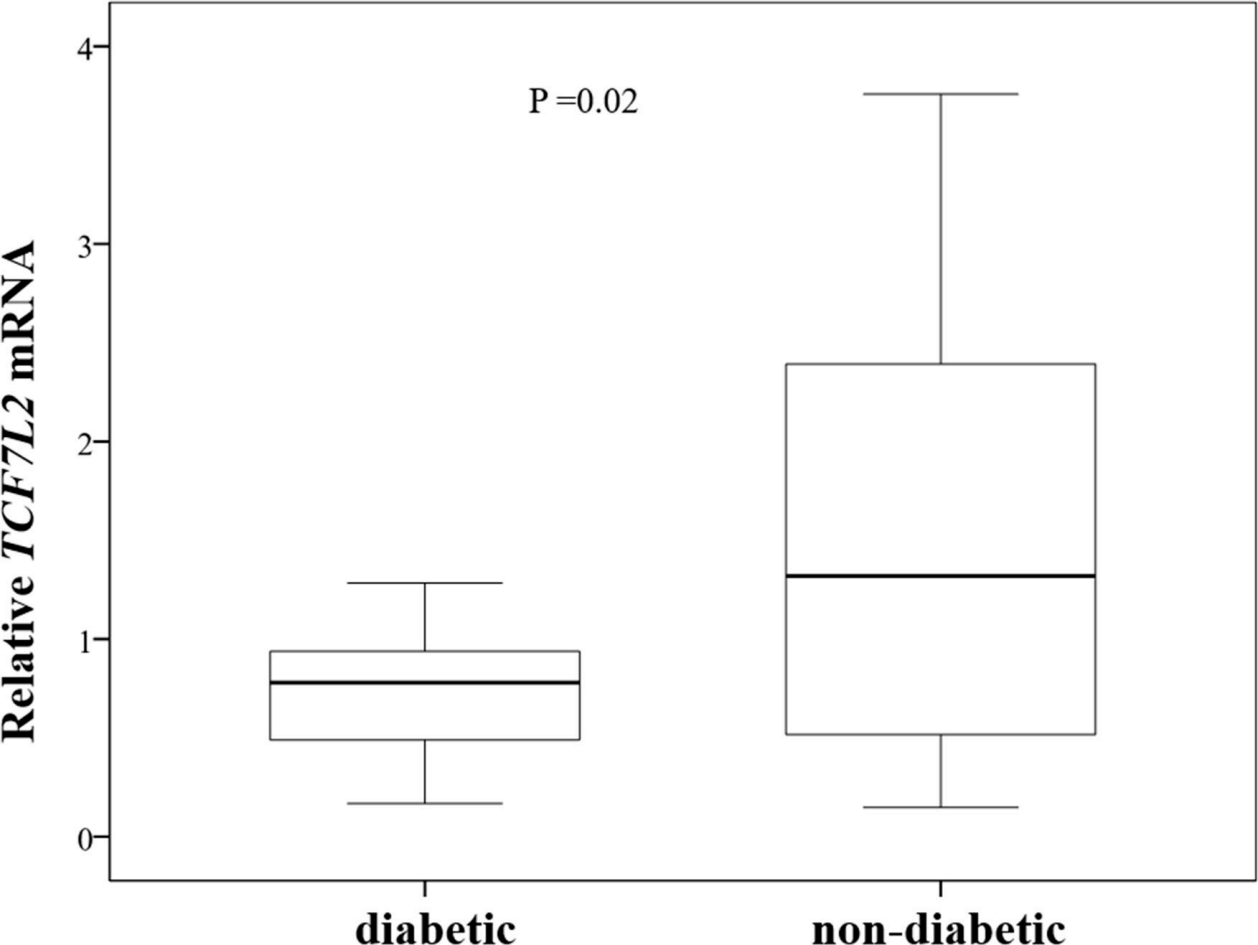
Comparison of the variation in the expression of *TCF7L2* (relative *TCF7L2* mRNA) between diabetic and non-diabetic patients.

Assessment of relative expression in the subgroups regarding the presence of T2DM showed an average increase of 48% (mean 1.48; SD 1.07) in non-diabetic patients, and an average decrease of 27% (mean 0.73; SD 0.33) in those with T2DM one year after surgery. However, such variation was statistically significant only for the diabetic group (p = 0.021). The variation in gene expression pattern differed between the two groups (Fig 2), with a p-value = 0.02.

Regarding the postoperative *TCF7L2* gene expression, the subgroups of diabetic and non-diabetic patients were assessed using the same categorical variable, namely those with an increase or decrease in relative gene expression (values higher or lower than 1). Diabetic patients were more likely to have reduced gene expression than non-diabetic patients (81.88% versus 40%), and this difference almost reached statistical significance (p = 0.051).

**Fig 3:**
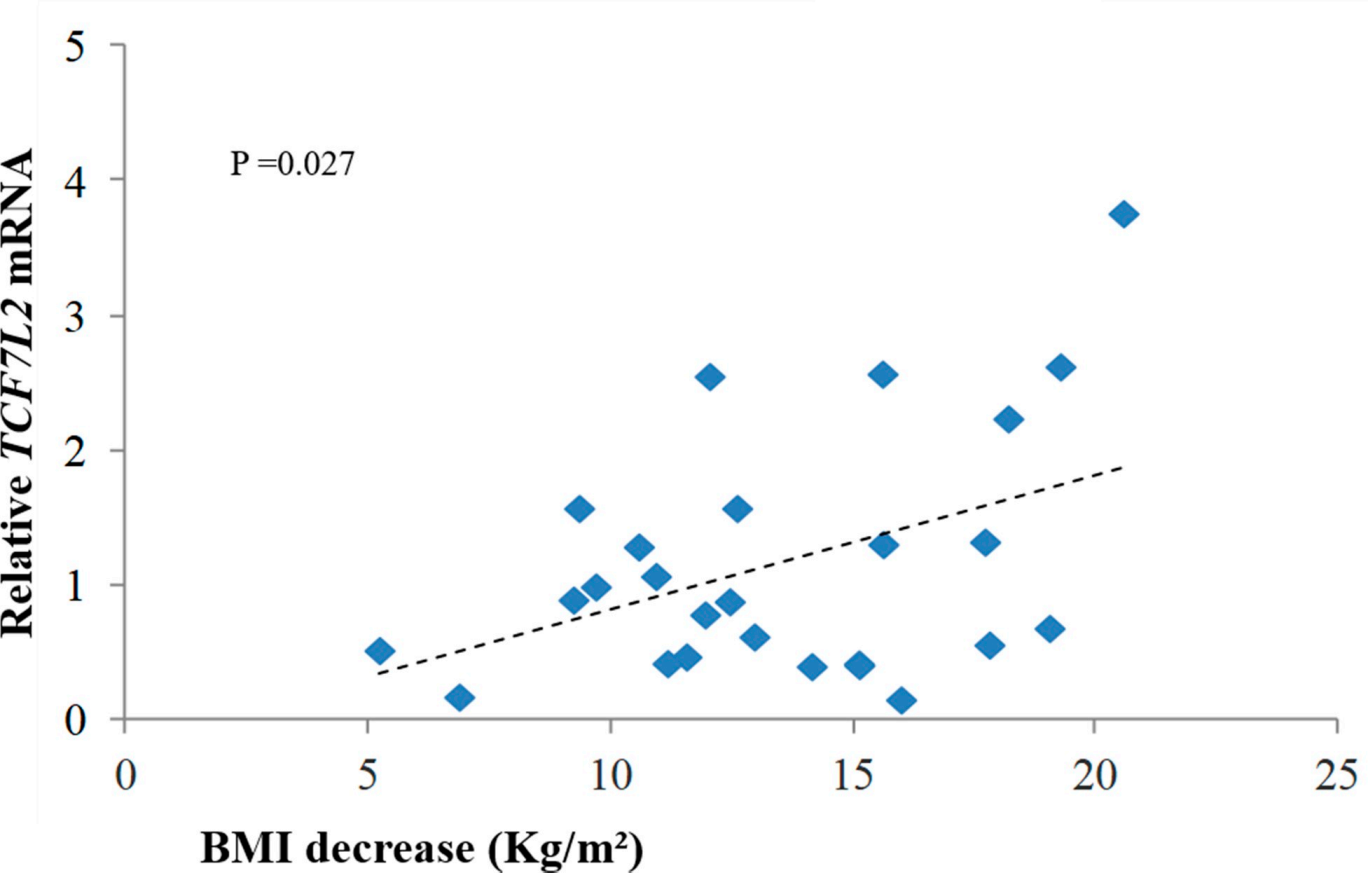
Linear regression analysis: decrease in BMI one year after surgery x genetic expression of *TCF7L2* (relative *TCF7L2* mRNA).

A linear regression model was used to assess the relationship between BMI decrease and relative expression of *TCF7L2* after bariatric surgery. As shown in Fig 3, there was a positive correlation between a greater decrease in BMI and an increase in relative expression, which was also statistically significant (p = 0.027). However, no statistically significant relationship was obtained with the use of a similar regression model for assessing the diabetic and non-diabetic subgroups.

## Discussion

The relationship between BMI and delta-Ct of gene expression in the preoperative period (Fig 1A) showed a positive correlation (0.037), indicating that the higher the BMI, the lower the genetic expression. When the patients were divided into two subgroups (diabetic and non-diabetic patients) and despite a p-value > 0.05, a divergent pattern was observed, with diabetics tending to show a negative correlation, while the opposite occurred with non-diabetic patients. These findings corroborate the results obtained by Hindle et al., who assessed *TCF7L2* gene expression in the preoperative period of class III obese patients undergoing bariatric surgery using delta-Ct as a measure of gene expression. However, no statistically significant difference was detected in the general population in the referred study, but only in the assessment of the subgroups (diabetic and non-diabetic) [7].

The differences between these findings and those exhibited in the present study can be due to the sample size and the type of material analyzed; in the study by Hindle et al., gene expression assessment was based on liver fragments and omental fat, while the present study was based on peripheral blood [7].

In using the abovementioned methodology, in the postoperative period (Fig 1B), it was noticed that the positive correlation between delta-Ct and BMI observed in the preoperative period was similar, and also statistically significant (p = 0.007). However, when analyzing the subgroups, the relationship between delta-Ct and BMI in diabetic patients was reversed after surgery, showing the same pattern of non-diabetic patients. The correlation was only statistically significant in the non-diabetic group (p = 0.039).

These findings suggest the hypothesis that increased weight leads to a decrease in *TCF7L2* gene expression. However, diabetic patients do not follow this pattern, which is intriguing as it suggests that the onset of T2DM after weight gain in a subgroup of individuals, as well as the resolution of T2DM after weight loss, are associated to inadequate regulation of *TCF7L2* expression due to BMI variations.

When analyzing relative expression, no alteration was observed after surgery in the assessed population. When the patients were separated into diabetic and non-diabetic, a heterogeneous distribution of the genetic expression alterations was observed in diabetic patients one year after surgery. The average relative expression was 0.725, while it was 1.478 in the non-diabetic group. However, there was only significant alteration in gene expression in the subgroup of diabetic patients (p = 0.021), while there was no statistically significant difference in non-diabetics due to the high heterogeneity of the alterations, despite an average increase of 47.8%.

The gene expression alterations observed after bariatric surgery corroborate the hypothesis that the relationship between *TCF7L2* gene expression and BMI in diabetic patients exhibits different patterns in relation to non-diabetic individuals. Despite the various alterations in *TCF7L2* expression, especially in non-diabetic individuals, the tendency of diabetic patients to maintain or reduce gene expression after surgery (and consequently weight loss and resolution of T2DM) was relatively homogeneous and constant, with a statistically significant difference between the two subgroups (p = 0.02).

*TCF7L2* expression also increased with greater decrease in BMI, and this relationship was statistically significant (p = 0.027). However, the subgroup analysis failed to establish any significant differences, probably due to the sample size.

We raise the hypothesis that one mechanism of T2DM resolution in the postoperative period of bariatric surgery is the modulation of *TCF7L2* expression, which is probably related to weight loss. The findings of the current study corroborate this hypothesis, showing different *TCF7L2* expression patterns in the preoperative period between the two subgroups, as well as different patterns of gene expression variations after bariatric surgery. Also, there is a relationship between a decrease in BMI and an increase in *TCF7L2* expression in the general population.

Kaminska et al. showed no gene expression alterations in fat and liver tissue before and one year after bariatric surgery, but demonstrated an alteration in the distribution of mRNA variants produced by the gene [8]. Furthermore, the *TCF7L2* gene plays an important role in regulating adipose tissue development and function, and this is probably associated with the increase in insulin resistance [14]. In the present study, part of the observed heterogeneity of the gene expression alterations can be explained by the modulation of the alternative splicing, as depending on where the exons are inserted, the resulting protein can be a strong stimulant or even an inhibitor of the Wnt pathway. In other words, even though 80% of the diabetic patients presented decreased *TCF7L2* expression in the postoperative period, it is not possible to rule out a decrease of inhibiting transcripts and maintenance or even an increase of stimulating transcripts, resulting in increased gene activity and better glucose control. A few studies have demonstrated the possible role of alternative splicing in the physiopathogenesis of T2DM [15,16].

In an experimental study on pancreatic islets, Zhou et al. demonstrated that there was a reduction of proinsulin expression in both cells where deletion occurred and in those where there was increased *TCF7L2* expression. We believe that (just like in the current study) the variable expression of activating and inhibiting isoforms, which together promote an increase in expression, do not necessarily result in an increased gene effect. This would be determined by the complex interaction between such transcripts [9].

Since there was no assessment of produced mRNA variants in the present study, but rather an overall assessment of expression, such a hypothesis cannot be corroborated, and should be analyzed in further studies. However, in this study it was demonstrated that the outcomes produced by bariatric surgery lead to alterations in *TCF7L2* gene expression, and that both the decrease in BMI and the presence of T2DM modulate such alterations.

The mechanism through which obesity modulates gene processing and expression can be related to possible epigenetic mechanisms, such as the methylation of regions adjacent to the gene [10]. An experimental study evidenced that high-fat diets can induce the *TCF7L2* promoter methylation, consequently interfering in its genetic expression [17]. Likewise, alterations in the expression of *TCF7L2* transcripts in the liver, as well as in other non-pancreatic tissues and pancreatic pericytes, can also be associated to the physiopathogenesis of T2DM [11, 18, 19].

Also, gene expression in previous studies was not assessed in peripheral blood. Peripheral blood was used in the present study because gene expression in serum concentration might also be associated to glucose control and because of its easy collection, which makes it easier to obtain new samples throughout the follow-up. As far as we know, this is the first study that shows altered *TCF7L2* gene expression in serum after bariatric surgery, as well as its potential associations with diabetes and decrease in BMI.

Due to the small sample size, the current study cannot confirm if the serum *TCF7L2* gene expression might have a predictive value regarding postoperative weight loss and/or T2DM remission in patients undergoing bariatric surgery. However, our results encourage the development of future studies on this field to investigate the hypothesis of the *TCF7L2* expression as a predictor of the effectiveness of bariatric surgery.

## Conclusion

Taking the results of the present study into consideration, it is possible to conclude that there was a negative correlation between BMI and *TCF7L2* expression, however we have also found an opposite relationship between BMI and gene expression among diabetic patients after bariatric surgery, namely there was a reduction of *TCF7L2* expression with the weight loss produced by the surgery. Furthermore, it was demonstrated that the BMI decrease is associated to the increased relative expression of *TCF7L2* among the general population.

Further studies are needed in order to assess the variations of *TCF7L2* isoforms expression and its relationship with weight loss, as well as to analyze the behavior of these isoforms in T2DM patients and their possible association with the resolution of the disease.

## Supporting information

S1 Dataset Individual patient data. (XLSX)
